# Dissecting adult plant resistance to stem rust through multi-model GWAS in a diverse barley germplasm panel

**DOI:** 10.3389/fpls.2025.1681398

**Published:** 2025-10-08

**Authors:** Yuliya Genievskaya, Akerke Maulenbay, Shynbolat Rsaliyev, Saule Abugalieva, Aralbek Rsaliyev, Alibek Zatybekov, Yerlan Turuspekov

**Affiliations:** 1 Laboratory of Molecular Genetics, Institute of Plant Biology and Biotechnology, Almaty, Kazakhstan; 2 Laboratory of Phytosanitary Safety, Research Institute of Biological Safety Problems, National Holding “QazBioPharm”, Gvardeisky, Zhambyl, Kazakhstan; 3 Laboratory of Cereal Crops, Kazakh Research Institute of Agriculture and Plant Growing, Almalybak, Almaty, Kazakhstan

**Keywords:** *Hordeum vulgare* L., disease resistance breeding, quantitative trait loci (QTLs), *Puccinia graminis* f. sp. *tritici*, gene expression analysis

## Abstract

**Introduction:**

Stem rust (SR), caused by *Puccinia graminis* f. sp. *tritici* (*Pgt*), remains a major threat to global barley production, particularly in regions with conducive environments and evolving pathogen populations. Despite progress in understanding seedling resistance, adult plant resistance (APR) to SR remains underexplored in diverse barley germplasm. This study aimed to dissect the genetic architecture of APR to SR in a panel of diverse origins of two-row spring barley using a genome-wide association study (GWAS).

**Methods:**

A total of 273 barley accessions were evaluated for APR to SR in two distinct environments in Kazakhstan. Phenotypic data were combined with high-density SNP genotyping to perform GWAS using five statistical models (GLM, MLM, MLMM, FarmCPU, and BLINK). Population structure and kinship were accounted for to identify robust marker-trait associations (MTAs), followed by haplotype-based QTL delineation. Transcriptomic data from 16 barley tissues were used to identify candidate genes within major QTL regions. Substantial phenotypic variation in SR severity was observed across environments.

**Results:**

A total of 204 MTAs were identified, among which 96 were stable across models, resulting in 19 model-stable QTLs spanning all seven barley chromosomes. Six QTLs co-localized with known SR-resistance QTLs and genes, including *Rpg1* and *Rpg6*. *Q_rpg_7H.1* (coinciding with *Rpg1*) was one of the strongest and most consistent QTL, harboring 42 highly expressed candidate genes. A novel major-effect QTL on chromosome 5H, *Q_rpg_5H.1* (3.5 – 9.9 Mb), not previously associated with known resistance loci, contained 10 highly expressed genes grouped into three co-expression clusters, including WRKY transcription factors and PR-5 proteins.

**Conclusion:**

This study provides new insights into the complex, multilayered genetic control of SR resistance in barley. The discovery of both known and novel QTLs offers valuable targets for marker-assisted selection and lays the foundation for breeding durable SR-resistant barley adapted to diverse agroecological conditions.

## 1 Introduction

Barley (*Hordeum vulgare* L.) is one of the most important cereal crops in Kazakhstan, occupying a significant share of the arable land and contributing substantially to the national agricultural output. It is predominantly grown in rain-fed agricultural zones, where its exceptional adaptability to a wide range of abiotic stresses – including drought, soil salinity, and low temperatures – makes it a reliable crop under the region’s often harsh and variable climatic conditions (Newton et al., 2011). These attributes are particularly important for Kazakhstan, where environmental limitations frequently constrain agricultural productivity. As the second most widely cultivated cereal crop after wheat (Bureau of National statistics, 2025), barley plays a crucial role in ensuring national food security and rural livelihoods. It serves multiple purposes: as a staple component of livestock feed, a valuable raw material for the malting and brewing industries, and a food source for human consumption, particularly in regions with limited wheat availability (Verma et al., 2022).

Among the biotic stresses affecting barley, rust diseases, including stem, leaf, and stripe rusts caused by fungal pathogens, are among the most economically damaging (Paulitz and Steffenson, 2010). However, stem rust (SR) ranks among the most devastating (Dill-Macky et al., 1991). Barley is susceptible to two SR pathogens: *Puccinia graminis* f. sp. *tritici* Eriks. and E. Henn. (*Pgt*), also known as the wheat SR fungus, and *P. graminis* f. sp. *secalis* Eriks and E. Henn. (*Pgs*), or the rye SR fungus. Of these, *Pgt* is a significantly greater threat in most major barley production regions, and historically, it has posed a major threat to barley production worldwide (Harder and Legge, 2000).

The average yield losses of barley due to barley SR often reach 10–25% (Murray and Brennan, 2010; Al-Abdallat et al., 2017; Çelik Oğuz and Karakaya, 2021). Recurrent *Pgt* epidemics are reported in various regions and often result in substantial yield losses up to 60% in susceptible cultivars and lower grain quality (Steffenson et al., 2017). A comparative study showed that susceptible barley cultivars experienced yield reductions of up to 58% during *Pgt* epidemics in Australia (Dill-Macky et al., 1991). In the Great Plains of the USA and Canada, *Pgt* epidemics have caused significant yield losses, exceeding 50%, along with declines in grain quality (Steffenson, 1992). The emergence of *Pgt* race TTKSK (Ug99) in Uganda in 1999 triggered a wave of concern in East Africa (Babiker et al., 2015). Barley SR epidemics caused by the Ug99 race of *Pgt* in Kenya caused a significant threat to barley production (Mwando et al., 2012). SR re-emerged in Europe in 2013, affecting wheat in Germany (Olivera Firpo et al., 2017) and later appearing in southern Denmark, eastern Sweden, and the UK (Lewis et al., 2018), followed by a major outbreak in Sicily, Italy, in 2016 (Bhattacharya, 2017). Since then, SR has been observed annually on wheat, barley, and rye in specific areas of Sweden (Kjellström, 2021). Barley SR epidemics in Kazakhstan are currently poorly described, but recent studies on *Pgt* races on wheat in the region offer valuable insights into the broader epidemiological landscape, suggesting a potential for significant threat to barley. From 2015 to 2019, severe wheat SR epidemics impacted northern Kazakhstan and western Siberia (Olivera et al., 2022). Analysis of 51 *Pgt* samples from Kazakhstan between 2015 and 2017 revealed 112 diverse races with similarities to races in Siberia, suggesting a shared epidemiological region and indicating spore inflow from the west (Olivera et al., 2022). In total, over 1900 cultivated barley accessions from across the globe were extensively evaluated, revealing that more than 95% were susceptible to TTKSK (Ug99) (Steffenson et al., 2017). The widespread susceptibility of cultivated barley germplasm to a single *Pgt* pathotype represents an unusually severe and dangerous threat to global food security. Given the re-emergence of *Pgt* epidemics in Europe and East Africa, understanding and deploying SR resistance in barley has global implications for food security under changing climate scenarios.

Nine SR resistance genes have been identified in barley: *Rpg1* (chromosome 7H, encodes a receptor kinase-like protein with two tandem protein kinase domains) (Brueggeman et al., 2006), *Rpg2* (chromosome 2H) (Case et al., 2018) and *Rpg3* (chromosome 5H) (Case et al., 2018), *rpg4* (chromosome 5H) (Steffenson et al., 2009) and *Rpg5* (chromosome 5H) (Sun et al., 1996), the RMRL (*rpg4/Rpg5*, chromosome 5H) complex (Brueggeman et al., 2006; Steffenson et al., 2009), *Rpg6* (*Hordeum bulbosum* introgression, chromosome 6H) (Fetch et al., 2009), *Rpg7* (chromosome 3H) (Henningsen et al., 2021), *RpgU* (unmapped) (Fox and Harder, 1995), and *rpgBH* (unmapped) (Steffenson et al., 1984). Although resistance conferred by the *Rpg1* gene has provided durable protection since the 1940s (Steffenson, 1992), recent emergent races such as QCCJB and TTKSK (Ug99) have demonstrated virulence to this and other resistance genes (Roelfs et al., 1993; Jin et al., 1994; Pretorius et al., 2000). The *rpg4*-mediated resistance, although highly effective against TTKSK, is temperature sensitive and acts recessively, making it challenging to incorporate into elite cultivars (Jin et al., 1994; Sun et al., 1996). The most effective immediate strategy for breeding SR-resistant barley involves combining the *Rpg1* gene with *rpg4/Rpg5* (Sallam et al., 2017). This genetic pyramid would safeguard the crop against the dominant virulence types found in the *Pgt* population. Additionally, genes *Rrr1* and *Rrr2* have been identified as important factors for pyramiding *Rpg1* and RMRL resistance genes in barley (Sharma Poudel et al., 2018). However, the identification of additional resistance loci remains a high priority, particularly those conferring durable, adult plant resistance (APR), which has been shown to offer broader and more sustainable protection compared to race-specific seedling resistance (Kolmer, 1996; Martinez et al., 2001).

Genome-wide association studies (GWAS) have emerged as a powerful approach to dissect the genetic basis of complex traits of barley (Alqudah et al., 2020), including disease resistance (Dubey and Mohanan, 2025), by leveraging the natural genetic variation present in diverse germplasm collections. However, the number of GWAS studies for SR resistance of barley is very limited. Unlike biparental mapping, GWAS uses existing diversity panels, enabling broader allele detection and finer mapping resolution due to historical recombination events. A key factor in the success of GWAS is the availability of high-density single-nucleotide polymorphism (SNP) markers, which provide genome-wide coverage and enable precise localization of trait-associated loci. GWAS of barley accessions grown in Kazakhstan have identified loci associated with critical agronomic traits, such as flowering time and plant architecture (Genievskaya et al., 2024), grain yield (Genievskaya et al., 2025), and grain quality (Genievskaya et al., 2022). As for the resistance to fungal diseases among cereal crops in Kazakhstan, previous GWAS efforts have successfully identified SNPs and QTLs associated with resistance to powdery mildew (Genievskaya et al., 2023) and SR (Turuspekov et al., 2016) in barley, showcasing the potential of this method for local breeding programs.

Despite the identification of several SR resistance genes and loci, the understanding of APR in diverse barley germplasm remains limited, particularly in the context of Kazakhstan and Central Asia. This study aimed to identify genetic loci associated with APR to SR in a diverse barley panel using multiple GWAS models, with a focus on uncovering candidate genes for durable resistance applicable to Kazakhstan and beyond.

## 2 Materials and methods

### 2.1 Germplasm material and SNP genotyping

A total of 273 spring two-row barley accessions, originating from the USA, Kazakhstan, Europe, Africa, and the Middle East (**Supplementary Table 1**), were cultivated under field conditions at the Research Institute of Biological Safety Problems (RIBSP; southern Kazakhstan, 43.576476° N, 75.213618° E) in 2024 and Kazakh Research Institute of Agriculture and Plant Growing (KRIAPG; southeastern Kazakhstan, 43.229402° N, 76.699168° E) in 2025. This barley panel was previously utilized for studies on adaptability and yield-related traits (Genievskaya et al., 2024, 2025). Genotyping was performed using the 50K Illumina Infinium iSelect SNP array (Bayer et al., 2017) (TraitGenetics GmbH, Gatersleben, Germany). Genotyping results were used in the analysis of population structure, linkage disequilibrium (LD), and further GWAS analyses. SNP physical positions were retrieved from the Morex v3 reference genome (The Triticeae Toolbox – Barley, 2025).

### 2.2 Evaluation of resistance to SR

To simulate SR epiphytotics in RIBSP, field plots were artificially inoculated with a virulent composite of *Pgt* races. These isolates were originally collected from Kazakhstan’s spring wheat cultivars (Rsaliyev et al., 2020) and are maintained in the microorganism collection of RIBSP. Prior to inoculation, urediniospores were reactivated by heat-shock treatment at 50°C for 30 minutes (following the protocol of Rsaliyev and Rsaliyev, 2019). A suspension of urediniospores (**Supplementary Table 2**) was prepared in 3M™ Novec™ 7100 (3M, USA) and uniformly applied to seedlings using an airbrush spray gun (Revell GmbH, Germany) (Patpour et al., 2022). Inoculations were conducted at the seedling stage during evening hours (Roelfs et al., 1992), and irrigation was applied immediately afterward to ensure adequate humidity for spore germination and disease establishment. In KRIAPG, assessment of SR resistance was conducted under natural infection conditions, with epiphytotic development resulting from an adjacent infected winter wheat field.

Field trials in both RIBSP and KRIAPG were established using a randomized complete block design (RCBD) with two replications. Each genotype was planted in two-row plots, 1.5m in length, with 30cm spacing between rows and 40cm between plots. In RIBSP, to promote uniform disease pressure, susceptible spreader rows (mixture of highly susceptible local cultivars) were planted after every 10 test entries and also used as border rows surrounding the experimental area. Resistant and susceptible checks were included in each replication to validate the reliability of disease assessments. Meteorological and environmental data from both fields are presented in **Supplementary Table 3**.

In both environments, SR severity was assessed at the milky-waxy seed development stage by estimating the percentage of infection using a modified Cobb scale (Peterson et al., 1948). Infection types were classified into five categories: immune (I), resistant (R), moderately resistant (MR), moderately susceptible (MS), and susceptible (S) (Roelfs et al., 1992). Traditional scoring was converted into McNeal’s 9-point scale (McNeal et al., 1971) for GWAS.

In RIBSP, prior to harvest, phenological traits (heading and maturity dates, vegetation period) and agronomic parameters (flag leaf area, plant height, upper internode, and spike length) were recorded following CIMMYT protocols (Pask et al., 2012). After natural grain drying, plot yield and thousand kernel weight were assessed using the same methodology.

### 2.3 Population structure and association analysis

Population structure was assessed using pairwise kinship coefficients and principal component analysis (PCA). The kinship matrix was calculated with GAPIT v3 (Wang and Zhang, 2021) and visualized using the heatmap3 package, while eigenvalues and PCA results were plotted with ggplot2 in R.

Using GAPIT, five GWAS models – GLM, MLM, MLMM, BLINK, and FarmCPU – were utilized to identify stable marker-trait associations (MTAs) for SR resistance. PCA.total=3 was used in all GWAS models for the correction of population structure effect. Consistency of significant signals across these methods determined stability. A *P*-value threshold of 1.00E-4 was set to capture all potential associations, acknowledging that the standard threshold may miss true associations in studies with low-frequency variants or smaller populations due to insufficient statistical power (Fadista et al., 2016). Studies have shown that relaxing the p-value threshold can improve the detection of associations with small effects, thereby capturing a broader spectrum of true genetic signals (Chen et al., 2021).

### 2.4 QTLs identification and candidate genes analysis

To consolidate closely linked MTAs into distinct quantitative trait loci (QTLs), the critical linkage disequilibrium (LD) distance at R² = 0.2 was previously calculated for each chromosome (Genievskaya et al., 2025) and used as the merging threshold. Within each QTL region, the SNP showing the lowest P-value was designated as the lead or peak SNP. Haplotype structure and allele combinations within QTLs were visualized using the SRplot online platform (SRplot, 2025).

Candidate gene identification was carried out by aligning the physical positions of known *Rpg* barley genes with the identified QTL intervals. A physical map displaying positions of identified QTLs and mapped *Rpg* genes was generated using MapChart v2.3 (Voorrips, 2002). To identify protein-coding genes potentially related to SR resistance within QTL regions, four databases were used. IDs of genes located within QTL intervals were retrieved from EnsemblPlants (Yates et al., 2022). Their expression profiles were examined using BarleyExpDB (Li et al., 2023) and RNA-Seq datasets from 16 tissues/organs of the Morex cultivar (Mascher et al., 2017). Genes exhibiting expression levels above 100 TPM (transcripts per million) were considered strong candidates. Functional annotation of encoded proteins was performed using UniProt (The UniProt Consortium, 2025) and QuickGO (QuickGO, 2025), and gene expression patterns were visualized via the “heatmap3” package for R. To investigate patterns of gene co-expression across tissues and organs and identify functionally coherent gene modules, a weighted gene co-expression network of highly expressed genes (TPM > 100) was constructed using expression data from 16 tissues/organs (Mascher et al., 2017). A Pearson correlation threshold of *r* ≥ 0.6 was applied to retain biologically meaningful associations. The resulting correlation matrix was converted to an undirected weighted network using the igraph package for R. Gene clusters were detected using the Walktrap community detection algorithm (Pons and Latapy, 2006).

## 3 Results

### 3.1 Field assessment of SR resistance

**Figure 1:**
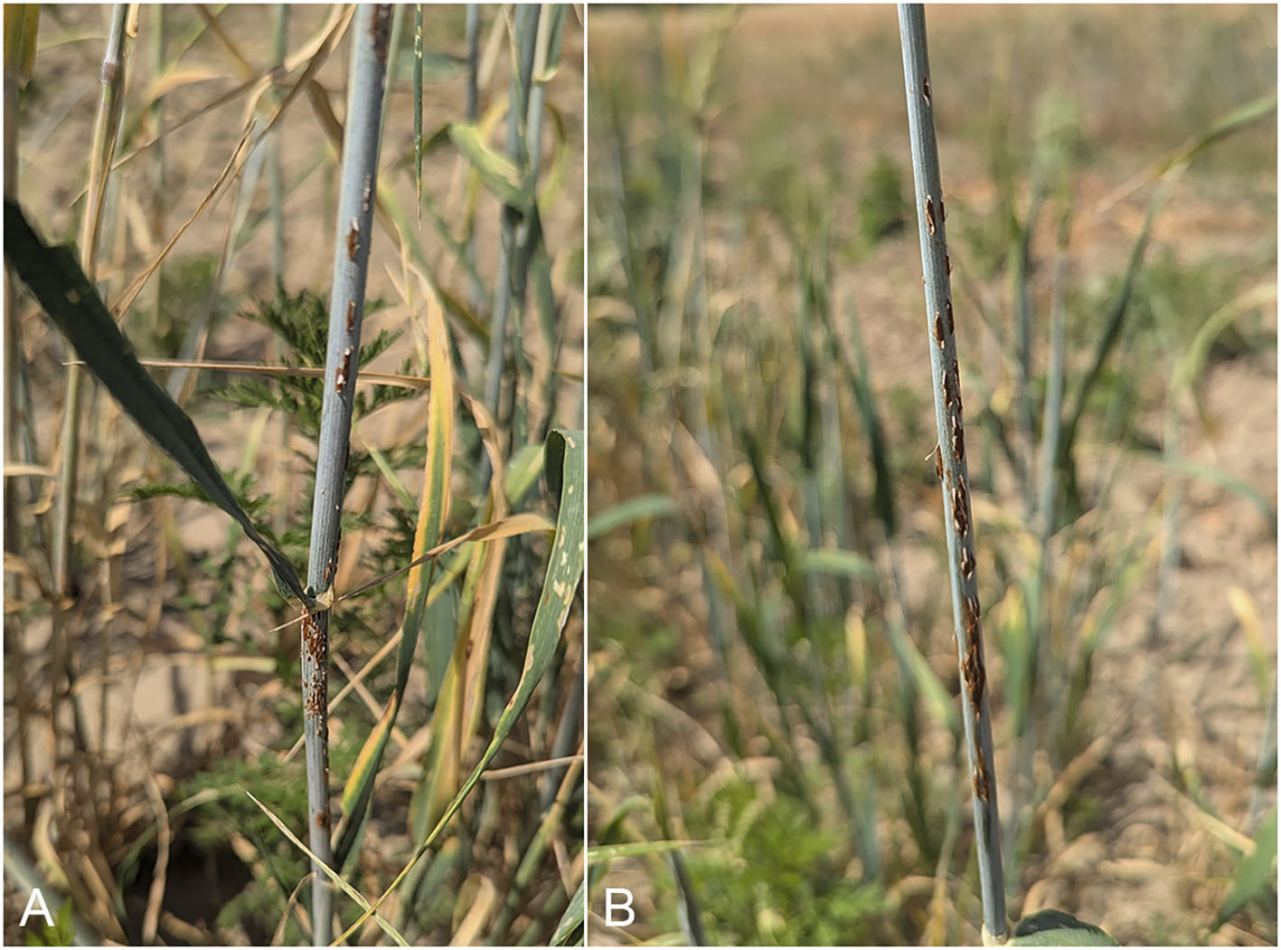
Barley cultivars susceptible to SR. Line QB_047 or 04WA-111-A from WA, USA, with 40S infection type in RIBSP **(A)**, and line QB_275 or PLD 139 from Poland with 70S infection type in KRIAPG **(B)**.

A total of 273 spring two-row barley accessions were evaluated for SR severity. On average, the severity at the milky-waxy seed development stage in RIBSP was 4.4 on a 9-point scale, corresponding to a score of 70–90MR using the classical IT scale, while in KRIAPG, the average score was 5.2, which corresponded to 10–30MS. Examples of susceptible cultivars from two environments are provided in **Figure 1**.

**Figure 2:**
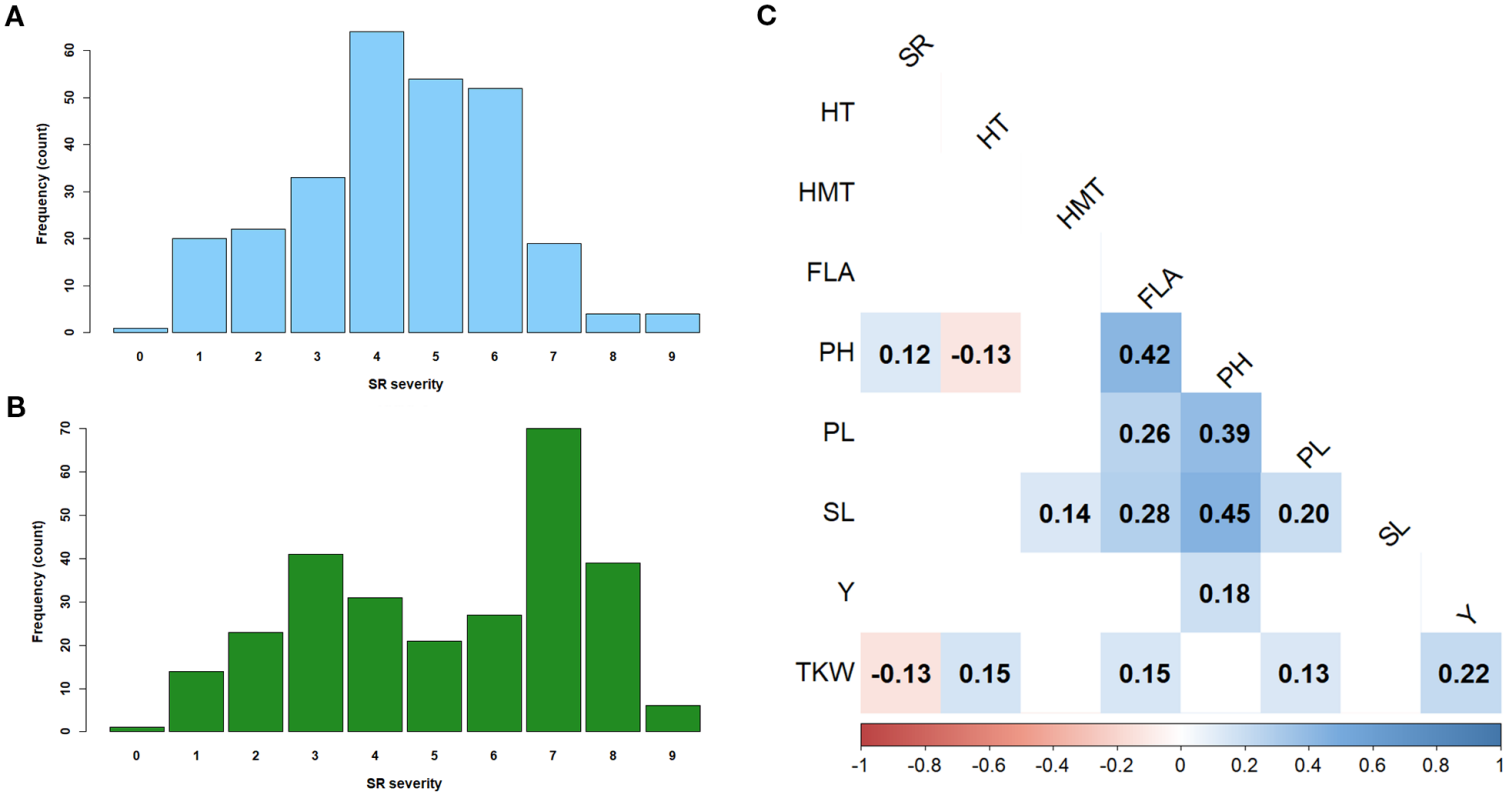
Frequency distribution of the barley accessions for stem rust resistance evaluated on a 9-point scale in RIBSP **(A)** and KRIAPG **(B)**; the correlation analysis among resistance to stem rust and key agronomic traits of barley in RIBSP **(C)**. SR, stem rust (*Pgt*) severity; HT, days from seedling emergence to heading; IT, infection type; HMT, days from heading to full grain maturity; FLA, flag leaf area; PH, plant height; PL, peduncle length; SL, spike length; Y, kernels yield per plot; TKW, 1000 kernels weight. Correlations with P-value < 0.05 are shown. Blue and red colors represent positive and negative correlations, respectively, with color intensity indicating the strength of the correlation; the scale bar at the bottom ranges *r*-values from −1 (strong negative correlation) to +1 (strong positive correlation).

In the RIBSP field, the barley collection displayed the full range of reactions to SR, from 0 (immune) to 9 (highly susceptible), with a standard deviation of 1.83 and a moderate CV of 40.6% (**Supplementary Table 4**). The distribution of SR severity scores on a 9-point scale (**Figure 2A**) approximated a normal curve, centered around scores 4 and 5, with the highest frequency observed at score 4 (64 counts). In the KRIAPG field, the collection also exhibited the complete range of SR responses (0–9), but with a higher standard deviation of 2.26 and a slightly greater CV of 43.15% (**Supplementary Table 4**). However, the distribution pattern in KRIAPG was bimodal, with prominent peaks at severity scores 3 and 7 (**Figure 2B**). In both environments, frequencies declined toward the extremes of the scale (0 and 9), suggesting that strong resistance or high susceptibility were less prevalent in the evaluated population.

Among the 273 barley accessions evaluated at RIBSP, accession QB_218 (breeding line ASHOS-168 from Kazakhstan) exhibited an immune (I, 0) reaction to the local *Pgt* pathogen. In addition, 20 accessions originating from Kazakhstan and the USA displayed a resistant (R, 1) type of reaction (**Supplementary Table 4**). The highest level of susceptibility (S, 9) was observed in two accessions from the USA: QB_054 (breeding line 04WA-123-G) and QB_065 (breeding line 04WA-109). At KRIAPG, accession QB_111 (breeding line 04AB093-A from the USA) also demonstrated an immune (I, 0) response to the local *Pgt* pathogen. Fourteen other accessions from the USA exhibited an R (1) type reaction, while four accessions originating from Kazakhstan, Europe, and the Middle East were found to be highly susceptible (S, 9). Considering the results across both environments, three accessions from the USA – QB_027 (04AB093-B), QB_111 (04AB093-A), and QB_112 (04AB016-A) – exhibited the highest average level of resistance, classified as R (0.5 – 1). Five accessions from the USA and three accessions from Kazakhstan demonstrated an average S (7.5) type of reaction.

The correlation analysis in RIBSP demonstrated a significant (P < 0.05) positive correlation between SR severity and PH, as well as a negative correlation between SR and TKW (**Figure 2C**). The remaining studied agronomical traits did not significantly correlate with SR severity.

### 3.2 Genotyping and population structure

SNP genotyping resulted in 44,040 markers, of which 31,834 polymorphic SNP markers were selected after filtering for a call rate > 0.9 and minor allele frequency (MAF) ≥ 0.05 (**Supplementary Table 5**). These SNPs were evenly distributed across the 7 barley chromosomes, with 1,445 lacking positional information. The total genome coverage was approximately 4.54 Gb, with chromosome 5H showing the highest SNP density (average spacing 137.2 Kb) and chromosome 4H the lowest (233.1 Kb) (**Supplementary Figure 1**). The filtered SNP set was used for population structure analysis and GWAS.

**Figure 3:**
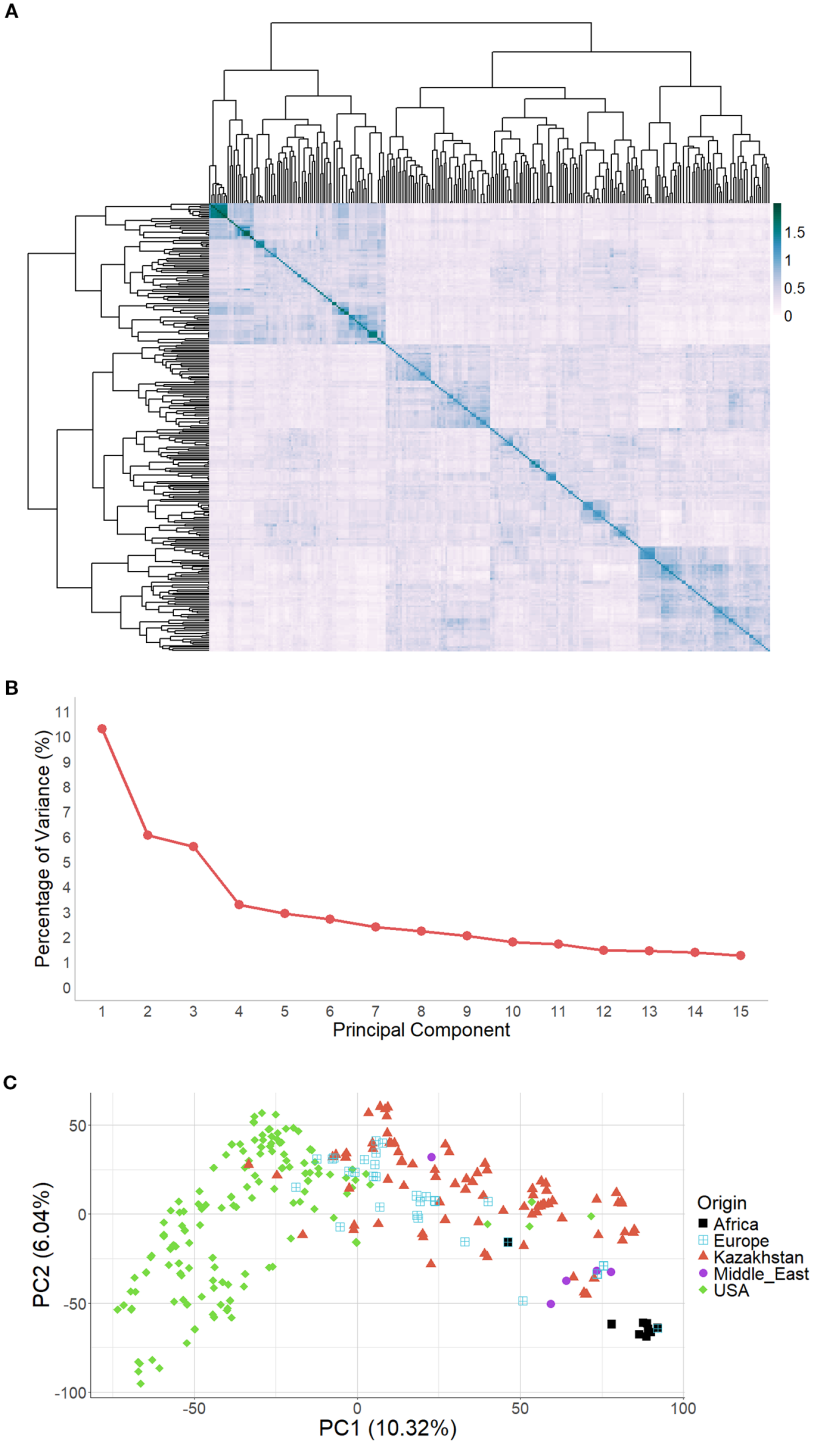
Population structure of barley germplasm based on 31,834 SNPs, including a heat map of the kinship matrix **(A)**, a scree plot of principal components **(B)**, and a PCA plot **(C)**.

The dendrogram derived from the kinship heatmap (**Figure 3A**) provided insights into the genetic structure of the barley population, revealing distinct clusters based on genetic relatedness. PCA showed that PC1, PC2, and PC3 explained 10.32%, 6.04%, and 5.59% of the total genetic variation, respectively (**Figure 3B**). The PCA plot (**Figure 3C**) displayed three partially overlapping clusters corresponding to germplasm from the USA, African barley accessions, and the remaining genotypes. This clustering pattern suggested the presence of at least two major groups – accessions from the USA and the remaining barley accessions. Additionally, smaller subclusters composed of closely related individuals were observed within these primary groups in the kinship heatmap.

### 3.3 GWAS and haplotype analysis

**Figure 4:**
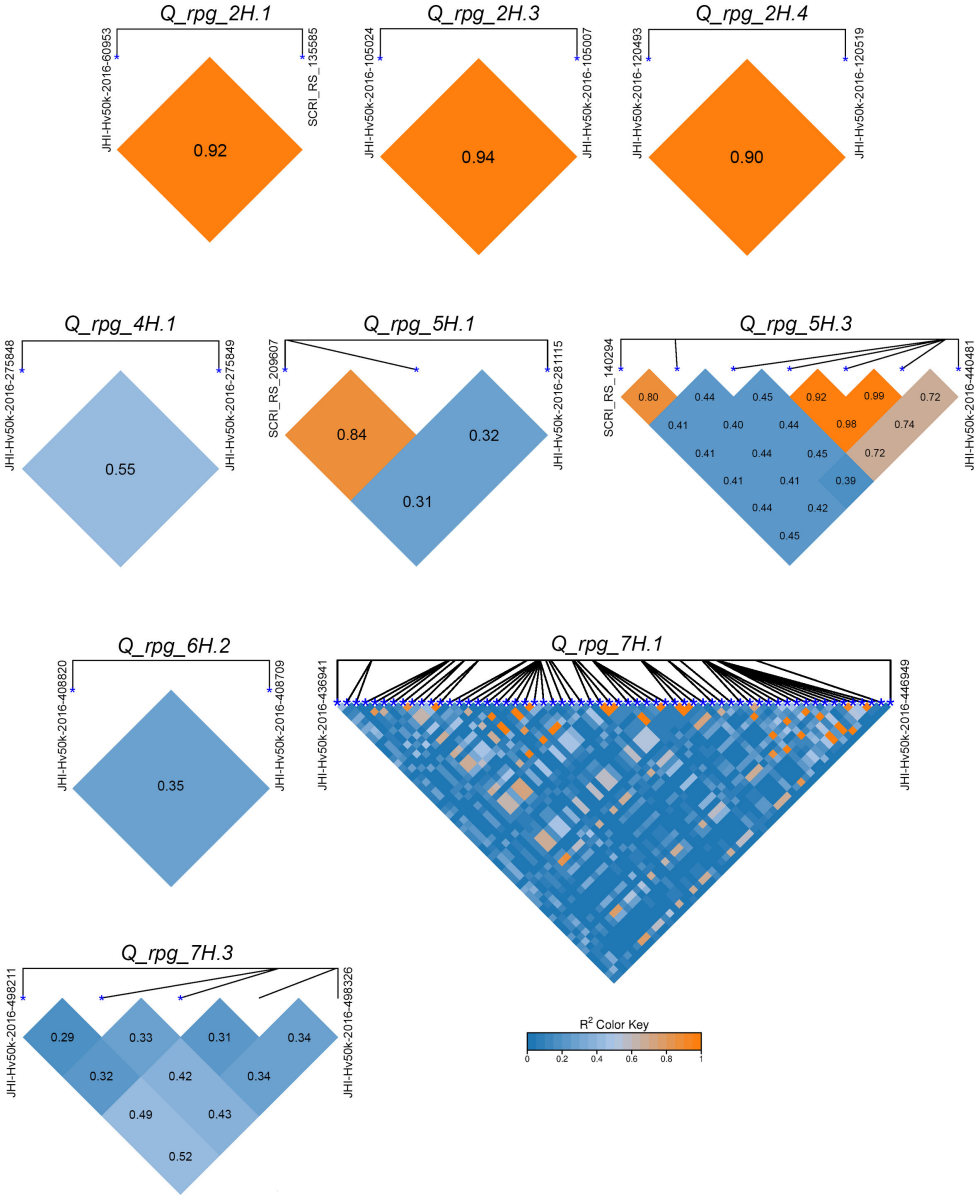
Linkage disequilibrium (LD) heatmaps for stem rust resistance QTLs. The color intensity, as indicated by the R2 color key, along with numerical values within the blocks, represents the strength of LD between marker pairs.

In total, 204 significant (P < 1.00E-4) MTAs for SR resistance were identified across the five models in two environments, including 62 MTAs for RIBSP and 142 from KRIAPG. The largest number of MTAs were identified with the GLM model (n = 111), followed by MLM (n = 44), FarmCPU (n = 30), BLINK (n = 14), and MLMM (n = 5). Among these, 96 MTAs were considered robust and stable, as they were consistently detected by at least two of five GWAS models. To consolidate overlapping signals, haplotype analysis was performed. MTAs located in close proximity and exhibiting R^2^ > 0.2 were grouped into single QTL intervals. This approach led to the identification of 19 model-stable QTLs associated with SR resistance. Among these, 9 QTLs were represented by multiple SNPs (ranging from 2 to 60) (**Figure 4**), while the remaining 10 were detected by single SNPs.

Detailed information regarding MTAs, their positions within QTLs, P-values, phenotypic values explained (PVE) values, and effects is provided in **Supplementary Table 6**. **Table 1** summarizes 19 QTLs for SR resistance identified across seven chromosomes.

**Table 1:** The list of stable QTLs for SR resistance identified using five GWAS models.

QTL	SNP	Chr	Pos	Effective GWAS model	Environment	Min *P*-value
*Q_rpg_1H.1*	JHI-Hv50k-2016-450275	1H	24,930,749	BLINK, MLMM	RIBSP	8.38E-05
*Q_rpg_2H.1*	JHI-Hv50k-2016-60953	2H	2,864,871	GLM, MLM, MLMM	KRIAPG	9.08E-05
	SCRI_RS_135585	2H	2,876,150	GLM, MLM, MLMM	KRIAPG	9.08E-05
*Q_rpg_2H.2*	JHI-Hv50k-2016-96261	2H	529,553,885	GLM, MLM	KRIAPG	5.45E-05
*Q_rpg_2H.3*	JHI-Hv50k-2016-105024	2H	561,314,845	FarmCPU, GLM, MLMM	KRIAPG	7.07E-09
	JHI-Hv50k-2016-105007	2H	561,317,792	FarmCPU, GLM, MLMM	KRIAPG	1.35E-05
*Q_rpg_2H.4*	JHI-Hv50k-2016-120493	2H	617,637,311	GLM, MLM	KRIAPG	5.15E-06
	JHI-Hv50k-2016-120519	2H	617,794,503	GLM, MLM	KRIAPG	5.15E-06
*Q_rpg_2H.5*	JHI-Hv50k-2016-128164	2H	630,897,103	BLINK, GLM, MLMM	KRIAPG	2.44E-05
*Q_rpg_2H.6*	JHI-Hv50k-2016-137128	2H	744,899,559	GLM, MLM	KRIAPG	3.53E-05
*Q_rpg_3H.1*	JHI-Hv50k-2016-225043	3H	695,553,560	BLINK, GLM, MLM, MLMM	KRIAPG	7.28E-05
*Q_rpg_4H.1*	JHI-Hv50k-2016-275848	4H	607,720,695	FarmCPU, GLM, MLMM	KRIAPG	1.70E-08
	JHI-Hv50k-2016-275849	4H	607,720,753	FarmCPU, GLM, MLMM	KRIAPG	1.22E-05
*Q_rpg_5H.1*	SCRI_RS_209607	5H	3,578,041	FarmCPU, MLMM	KRIAPG	6.21E-05
	JHI-Hv50k-2016-278508	5H	3,626,994	BLINK, MLMM	KRIAPG	4.75E-05
	JHI-Hv50k-2016-281115	5H	9,907,958	FarmCPU, MLM, MLMM	KRIAPG	2.86E-08
*Q_rpg_5H.2*	JHI-Hv50k-2016-303063	5H	416,597,383	GLM, MLM	KRIAPG	2.10E-06
*Q_rpg_5H.3*	SCRI_RS_140294	5H	453,313,031	GLM, MLM	KRIAPG	2.78E-05
	JHI-Hv50k-2016-310061	5H	453,485,876	GLM, MLM	KRIAPG	2.88E-05
	JHI-Hv50k-2016-440886	5H	454,345,120	GLM, MLM	KRIAPG	1.41E-07
	JHI-Hv50k-2016-440885	5H	454,345,196	Farm CPU, GLM, MLM	RIBSP	2.60E-05
	JHI-Hv50k-2016-440881	5H	454,345,338	Farm CPU, GLM, MLM	RIBSP	2.60E-05
	JHI-Hv50k-2016-440878	5H	454,345,443	Farm CPU, GLM, MLM	RIBSP	2.60E-05
	JHI-Hv50k-2016-440481	5H	454,386,257	BLINK, FarmCPU, GLM, MLM, MLMM	RIBSP	3.36E-09
*Q_rpg_6H.1*	JHI-Hv50k-2016-405915	6H	422,368,603	FarmCPU, GLM, MLM	KRIAPG	5.69E-05
*Q_rpg_6H.2*	JHI-Hv50k-2016-408820	6H	464,406,639	BLINK, GLM, MLM, MLMM	KRIAPG	7.28E-05
	JHI-Hv50k-2016-408709	6H	464,461,224	BLINK, GLM, MLM, MLMM	KRIAPG	7.28E-05
*Q_rpg_6H.3*	JHI-Hv50k-2016-434736	6H	561,746,668	GLM, MLM	KRIAPG	6.20E-05
*Q_rpg_7H.1*	JHI-Hv50k-2016-436941	7H	219,064	GLM, MLM	KRIAPG	9.87E-05
	JHI-Hv50k-2016-437076	7H	934,727	GLM, MLM	KRIAPG	5.29E-05
	JHI-Hv50k-2016-437077	7H	934,824	GLM, MLM	KRIAPG	2.31E-05
	JHI-Hv50k-2016-435062	7H	2,540,126	FarmCPU, GLM, MLM	KRIAPG	2.67E-07
	JHI-Hv50k-2016-435161	7H	2,549,430	BLINK, FarmCPU, GLM, MLM, MLMM	KRIAPG	4.67E-13
	JHI-Hv50k-2016-435177	7H	2,550,374	GLM, MLM	KRIAPG	5.07E-05
	JHI-Hv50k-2016-435274	7H	2,564,218	BLINK, GLM, MLM	KRIAPG	8.52E-06
	JHI-Hv50k-2016-437935	7H	2,682,155	FarmCPU, GLM, MLM	RIBSP	2.57E-05
	SCRI_RS_8079	7H	2,877,097	FarmCPU, GLM, MLM	RIBSP	1.25E-05
	JHI-Hv50k-2016-437307	7H	2,877,915	GLM, MLM	KRIAPG	3.06E-05
	JHI-Hv50k-2016-437598	7H	3,170,162	FarmCPU, GLM, MLM	RIBSP	6.27E-06
	JHI-Hv50k-2016-437597	7H	3,170,183	FarmCPU, GLM, MLM	RIBSP	6.27E-06
	JHI-Hv50k-2016-439043	7H	4,183,410	GLM, MLM	KRIAPG	3.64E-05
	JHI-Hv50k-2016-439082	7H	4,186,736	GLM, MLM	KRIAPG	4.29E-07
	JHI-Hv50k-2016-439213	7H	4,412,992	GLM, MLM	KRIAPG	7.80E-07
	JHI-Hv50k-2016-439375	7H	4,417,609	FarmCPU, GLM, MLM	RIBSP	7.47E-06
	JHI-Hv50k-2016-439464	7H	4,440,986	GLM, MLM	KRIAPG	4.29E-07
	JHI-Hv50k-2016-439341	7H	4,451,118	GLM, MLM	KRIAPG	4.29E-07
	JHI-Hv50k-2016-439340	7H	4,451,198	FarmCPU, GLM, MLM	RIBSP	4.29E-06
	JHI-Hv50k-2016-439309	7H	4,452,490	GLM, MLM	KRIAPG	2.89E-06
	JHI-Hv50k-2016-439545	7H	4,455,199	FarmCPU, GLM, MLM	RIBSP	7.47E-06
	JHI-Hv50k-2016-439559	7H	4,455,831	FarmCPU, GLM, MLM	RIBSP	7.47E-06
	JHI-Hv50k-2016-439565	7H	4,456,295	GLM, MLM	KRIAPG	8.08E-07
	JHI-Hv50k-2016-439567	7H	4,456,340	GLM, MLM	KRIAPG	6.05E-06
	BOPA1_1555-631	7H	4,580,292	GLM, MLM	KRIAPG	1.26E-05
	JHI-Hv50k-2016-439829	7H	4,641,549	GLM, MLM	KRIAPG	6.93E-06
	JHI-Hv50k-2016-439890	7H	4,686,449	FarmCPU, GLM, MLM	RIBSP	2.56E-05
	JHI-Hv50k-2016-440562	7H	5,134,684	GLM, MLM	KRIAPG	5.40E-06
	JHI-Hv50k-2016-440879	7H	5,165,768	FarmCPU, GLM, MLM	RIBSP	2.60E-05
	JHI-Hv50k-2016-440882	7H	5,165,886	FarmCPU, GLM, MLM	RIBSP	2.60E-05
	JHI-Hv50k-2016-440888	7H	5,234,237	FarmCPU, GLM, MLM	RIBSP	2.60E-05
	JHI-Hv50k-2016-441670	7H	5,554,752	GLM, MLM	KRIAPG	2.67E-07
	JHI-Hv50k-2016-441664	7H	5,555,314	GLM, MLM	KRIAPG	5.50E-05
	JHI-Hv50k-2016-441653	7H	5,555,883	GLM, MLM	KRIAPG	1.67E-05
	JHI-Hv50k-2016-441652	7H	5,555,965	GLM, MLM	KRIAPG	1.67E-05
	JHI-Hv50k-2016-441643	7H	5,556,522	GLM, MLM	KRIAPG	1.67E-05
	JHI-Hv50k-2016-441961	7H	6,572,207	FarmCPU, GLM, MLM	RIBSP	3.18E-05
	JHI-Hv50k-2016-441962	7H	6,572,420	FarmCPU, GLM, MLM	RIBSP	3.18E-05
	JHI-Hv50k-2016-441967	7H	6,573,267	FarmCPU, GLM, MLM	RIBSP	3.18E-05
	SCRI_RS_160297	7H	6,590,892	GLM, MLM	KRIAPG	3.85E-06
	JHI-Hv50k-2016-442556	7H	7,130,958	GLM, MLM	KRIAPG	6.95E-05
	JHI-Hv50k-2016-442574	7H	7,131,854	FarmCPU, GLM, MLM	RIBSP	6.95E-05
	JHI-Hv50k-2016-442878	7H	7,382,135	GLM, MLM	KRIAPG	6.95E-05
	SCRI_RS_230060	7H	7,846,242	GLM, MLM	KRIAPG	6.21E-05
	JHI-Hv50k-2016-443382	7H	7,894,665	GLM, MLM	KRIAPG	6.21E-05
	JHI-Hv50k-2016-443385	7H	7,894,775	GLM, MLM	KRIAPG	6.21E-05
	JHI-Hv50k-2016-443386	7H	7,894,836	GLM, MLM	KRIAPG	6.21E-05
	JHI-Hv50k-2016-443408	7H	7,911,072	GLM, MLM	KRIAPG	6.21E-05
	JHI-Hv50k-2016-443414	7H	7,911,233	GLM, MLM	KRIAPG	6.95E-05
	JHI-Hv50k-2016-443540	7H	8,150,854	GLM, MLM	KRIAPG	6.21E-05
	BOPA2_12_31411	7H	8,151,497	GLM, MLM	KRIAPG	6.21E-05
	JHI-Hv50k-2016-443531	7H	8,152,062	GLM, MLM	KRIAPG	6.21E-05
	JHI-Hv50k-2016-443528	7H	8,152,213	GLM, MLM	KRIAPG	6.21E-05
	JHI-Hv50k-2016-443527	7H	8,152,219	GLM, MLM	KRIAPG	6.21E-05
	JHI-Hv50k-2016-443525	7H	8,152,485	GLM, MLM	KRIAPG	6.21E-05
	JHI-Hv50k-2016-443524	7H	8,152,542	GLM, MLM	KRIAPG	6.21E-05
	JHI-Hv50k-2016-443515	7H	8,153,340	GLM, MLM	KRIAPG	6.95E-05
	JHI-Hv50k-2016-443502	7H	8,153,864	GLM, MLM	KRIAPG	2.55E-05
	JHI-Hv50k-2016-443689	7H	8,964,705	FarmCPU, GLM, MLM	RIBSP	7.73E-05
	JHI-Hv50k-2016-446949	7H	11,788,159	GLM, MLM	KRIAPG	7.04E-05
*Q_rpg_7H.2*	SCRI_RS_234502	7H	58,907,935	GLM, MLM	KRIAPG	2.55E-05
*Q_rpg_7H.3*	JHI-Hv50k-2016-498211	7H	584,358,525	GLM, MLM, MLMM	KRIAPG	3.13E-05
	JHI-Hv50k-2016-498293	7H	584,388,608	GLM, MLM, MLMM	KRIAPG	3.13E-05
	JHI-Hv50k-2016-498295	7H	584,388,714	GLM, MLM, MLMM	KRIAPG	3.13E-05
	JHI-Hv50k-2016-498322	7H	584,395,482	GLM, MLM, MLMM	KRIAPG	3.13E-05
	JHI-Hv50k-2016-498326	7H	584,395,756	GLM, MLM, MLMM	KRIAPG	3.13E-05
*Q_rpg_7H.4*	JHI-Hv50k-2016-506060	7H	602,014,442	FarmCPU, GLM, MLM, MLMM	KRIAPG	4.03E-05

Among the identified loci, *Q_rpg_5H.3* and *Q_rpg_7H.1* were consistently detected by all five GWAS models across both environments. The QTLs *Q_rpg_3H.1*, *Q_rpg_6H.2*, and *Q_rpg_7H.4* were identified by four models, while nine QTLs were detected using three models, and two models supported the remaining 15 QTLs. The lowest *P*-values, below the Bonferroni-corrected threshold (P=1.57E-6), were observed for five QTLs: *Q_rpg_2H.3, Q_rpg_4H.1, Q_rpg_5H.1, Q_rpg_5H.3*, and *Q_rpg_7H.1* (**Table 1**), indicating a strong association with SR resistance. The QTL *Q_rpg_7H.1* encompassed the highest number of associated SNPs, totaling 60.

Based on PVE values, QTLs were classified into four categories: major-effect QTLs (PVE ≥ 0.10), moderate-effect QTLs (0.05 ≤ PVE < 0.10), minor-effect QTLs (0.01 ≤ PVE < 0.05), and very small-effect QTLs (PVE < 0.01). According to this classification, nine loci were categorized as minor-effect QTLs, three loci as moderate-effect QTLs, and seven loci as major-effect QTLs (**Supplementary Table 6**). The largest PVE was observed for *Q_rpg_5H.3* (0.3065) followed by *Q_rpg_7H.1* (0.3063). PVE values of the remaining major-effect QTLs varied from 0.1056 to 0.1215 (**Supplementary Table 6**).

**Figure 5:**
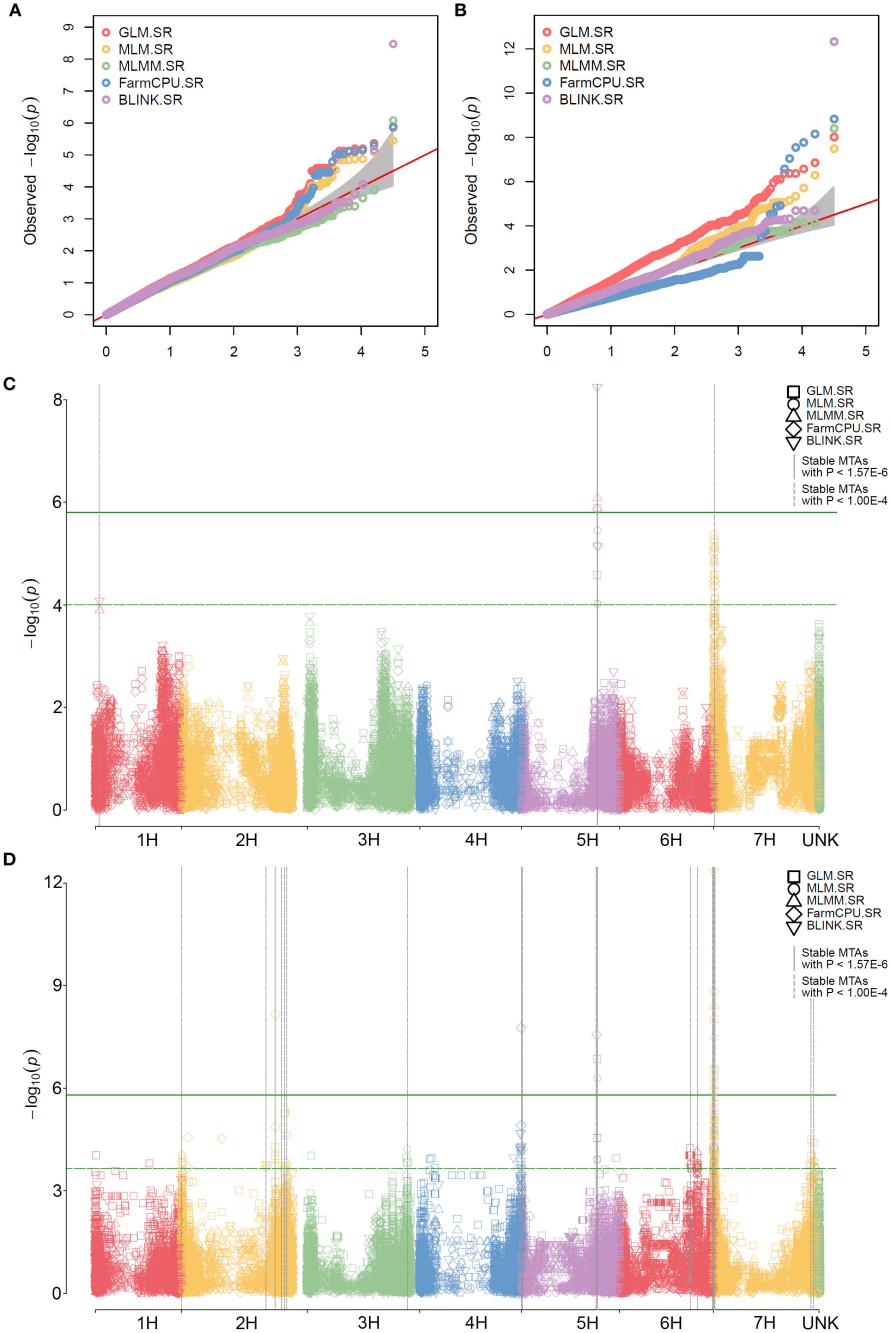
SNPs significantly associated with stem rust resistance in barley identified by GWAS with two or more models. Quantile-quantile plots with data from RIBSP **(A)** and KRIAPG **(B)**. Chromosome-wise Manhattan plots with data from RIBSP **(C)** and KRIAPG **(D)**. Associations stable across models are highlighted with vertical grey lines. The green solid horizontal line denotes a *P*-value of 1.57E-6 (Bonferroni); the green dashed horizontal line denotes a *P*-value of 1.00E-4.

The QQ plots for data from RIBSP and KRIAPG (**Figures 5A, B**) showed moderate inflation across all models, with clear deviations from the expected distribution in the upper tail, suggesting potential true associations with SR resistance. In the RIBSP dataset, the most significant peaks were located on chromosome 5H (**Figure 5C**), whereas in the KRIAPG dataset, the highest peaks were observed on chromosomes 2H, 4H, 5H, and 7H (**Figure 5D**), all surpassing the Bonferroni threshold. These findings support the presence of strong loci in the regions associated with SR resistance. Two major peaks on chromosomes 5H and 7H were consistently highly significant in both datasets.

**Figure 6:**
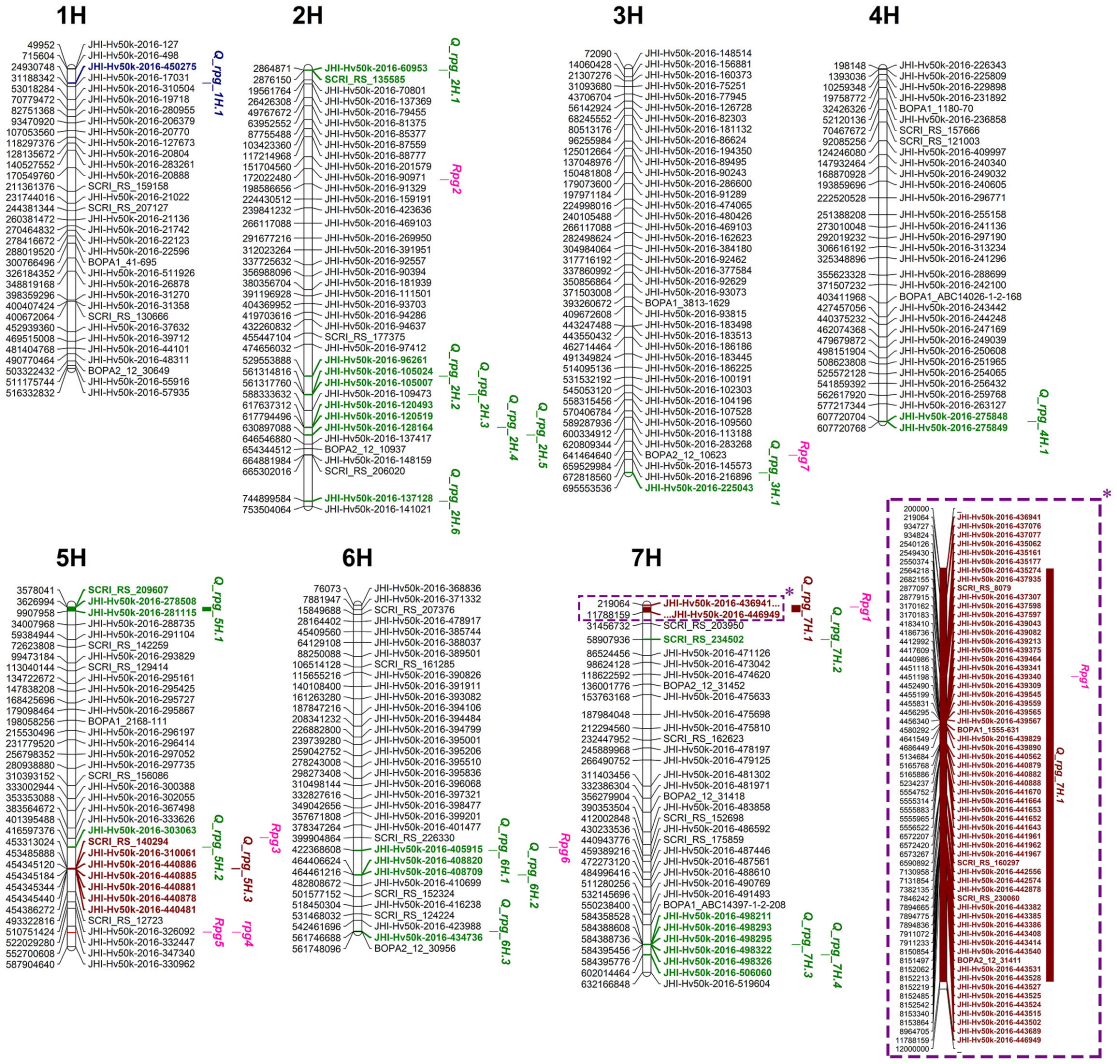
QTLs and their peak SNPs identified in the RIBSP dataset are shown in blue; QTLs from the KRIAPG dataset in green; and QTLs detected in both environments are indicated in brown. Known *Rpg* genes are highlighted in purple. An asterisk (*) denotes a close-up view of the corresponding segment on chromosome 7H.

The newly identified QTLs, along with known SR resistance genes (*Rpg1*, *rpg4*, and *Rpg5*), were mapped onto the barley genome across all seven chromosomes (**Figure 6**). The highest number of QTLs was detected on chromosome 2H (n = 6). The QTL *Q_rpg_7H.1* overlapped with the known *Rpg1* locus, indicating either a linked association or potential allelic variation. The remaining QTLs were located in genomic regions distinct from mapped *Rpg* loci, possibly representing novel resistance sources.

### 3.4 Candidate genes for stem rust resistance QTLs

Genetic positions of 19 QTLs mapped across all seven barley chromosomes were compared with positions of known *Rpg* genes and SR-resistance QTLs from the literature (**Table 2**).

**Table 2:** The list of reference genes and QTLs for newly identified SR resistance loci.

QTL	Position (interval)	Reference gene and/or QTL for SR resistance
*Q_rpg_1H.1*	24,930,749	–
*Q_rpg_2H.1*	2,864,871 – 2,876,150	–
*Q_rpg_2H.2*	529,553,885	–
*Q_rpg_2H.3*	561,314,845 – 561,317,792	–
*Q_rpg_2H.4*	617,637,311 – 617,794,503	(Czembor et al., 2022; Amouzoune et al., 2022)
*Q_rpg_2H.5*	630,897,103	–
*Q_rpg_2H.6*	744,899,559	–
*Q_rpg_3H.1*	695,553,560	–
*Q_rpg_4H.1*	607,720,695 – 607,720,753	–
*Q_rpg_5H.1*	3,578,041 – 9,907,958	–
*Q_rpg_5H.2*	416,597,383	–
*Q_rpg_5H.3*	453,313,031 – 454,386,257	(Czembor et al., 2022)
*Q_rpg_6H.1*	422,368,603	*Rpg6* (Henningsen et al., 2021)
*Q_rpg_6H.2*	464,406,639 – 464,461,224	(Turuspekov et al., 2016)
*Q_rpg_6H.3*	561,746,668	–
*Q_rpg_7H.1*	219,064 – 11,788,159	*Rpg1* (Henningsen et al., 2021)
*Q_rpg_7H.2*	58,907,935	–
*Q_rpg_7H.3*	584,358,525 – 584,395,756	–
*Q_rpg_7H.4*	602,014,442	(Czembor et al., 2022)

Six QTLs co-localized with genomic regions previously reported in the literature, supporting their relevance in barley SR resistance. The major resistance gene *Rpg1* was located within the *Q_rpg_7H.1* region, consistent with its known chromosomal position. The remaining 13 QTLs likely represent novel genetic factors associated with barley SR resistance.

**Figure 7:**
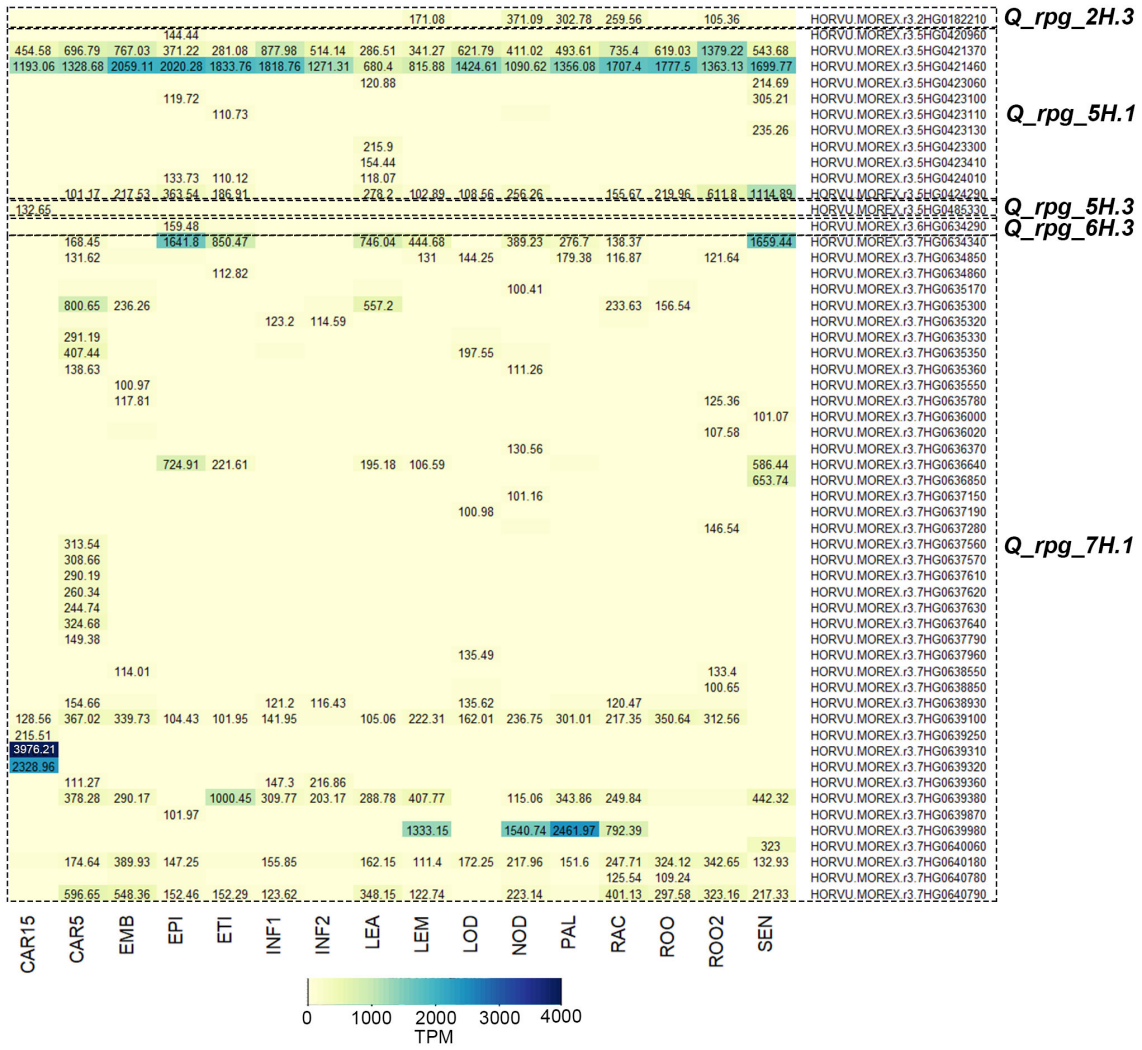
Expression heatmap (TPM > 100) of highly expressed candidate genes in QTLs associated with SR resistance at 16 plant developmental stages of barley. CAR15, Grain, bracts removed, 15 days post-anthesis; CAR5, Grain, bracts removed, 5 days post-anthesis; EMB, Embryos, 4 days dissected from germinating grains; EPI, 4-week-old epidermis; ETI, 10-day-old etiolated seedling; INF1, Young inflorescences, 5mm; INF2, Inflorescences, 1-1.5cm; LEA, 10cm shoot from the seedlings; LEM, Lemma, 6 weeks post-anthesis; LOD, Lodicule, 6 weeks post-anthesis; NOD, Six-leaf stage developing tillers; PAL, Palea, 6 weeks post-anthesis; RAC, Rachis, 5 weeks post-anthesis; ROO, 4-week-old root; ROO2, Roots from 10cm seedlings; SEN, 2-month-old senescing leaf.

Genes in stable QTL regions expressed in 16 barley tissues and organs of different developmental stages and organs were selected. A total of 531 candidate protein-coding genes with available expression data were located in 13 QTL regions (**Supplementary Table 7**). The remaining 6 QTLs were positioned in genomic regions not overlapping with coding barley genes. By filtering the low-expressed genes (TPM < 100), 56 highly expressed candidate genes were identified for five QTLs: *Q_rpg_2H.3* (n = 1), *Q_rpg_5H.1* (n = 11), *Q_rpg_5H.3* (n = 1), *Q_rpg_6H.3* (n = 1), and *Q_rpg_7H.1* (n = 42) (**Figure 7**).

The expression analysis revealed substantial variation in both transcript abundance and tissue specificity, allowing the prioritization of candidate genes potentially involved in SR resistance. Extremely high expression levels (TPM > 1000) were detected for eight genes located within two QTL regions.

In the *Q_rpg_5H.1* region, HORVU.MOREX.r3.5HG0421460 exhibited peak expression in nearly all analyzed tissues and organs, except for the 10cm shoot from seedlings (LEA, TPM=680.4) and lemma at six weeks post-anthesis (LEM, TPM=815.88). HORVU.MOREX.r3.5HG0421370 showed maximum expression in the roots of 10cm seedlings (ROO2) and high expression (TPM > 100) across all other organs and tissues. HORVU.MOREX.r3.5HG0424290 was highly expressed in the senescing leaf of 2-month-old plants (SEN), with relatively high expression in all other organs and tissues, ranging from 40.17 to 611.8 TPM (**Figure 7**). At the organ level, 9 out of 11 genes within *Q_rpg_5H.1* exhibited high expression (TPM > 100) in LEA, and 7 out of 11 in SEN.

In the *Q_rpg_7H.1* region, HORVU.MOREX.r3.7HG0634340 showed peak expression in the 4-week-old epidermis (EPI) and SEN (**Figure 7**). HORVU.MOREX.r3.7HG0639310 and HORVU.MOREX.r3.7HG0639320 were highly expressed in 15-day post-anthesis grains (CAR15), while HORVU.MOREX.r3.7HG0639380 peaked in 10-day-old etiolated seedlings (ETI). HORVU.MOREX.r3.7HG0639980 was most strongly expressed in LEM, developing tillers at the six-leaf stage (NOD), and palea at six weeks post-anthesis (PAL). Additionally, four genes – HORVU.MOREX.r3.7HG0639100, HORVU.MOREX.r3.7HG0639380, HORVU.MOREX.r3.7HG0640180, and HORVU.MOREX.r3.7HG0640790 – displayed consistently moderate to high expression across all analyzed tissues and developmental stages, with transcript levels ranging from 65.05 to 1000.45 TPM. Notably, 19 out of 42 genes are within *Q_rpg_7H.1* exhibited high expression (TPM > 100) in 5-day post-anthesis grains (CAR5).

In the *Q_rpg_2H.3* region, HORVU.MOREX.r3.2HG0182210 demonstrated high expression (TPM > 100) in LEM, NOD, PAL, 5 weeks post-anthesis rachis (RAC), and ROO2 (**Figure 7**). From the *Q_rpg_3H.1* region, HORVU.MOREX.r3.3HG0225930 was predominantly expressed in CAR15, while HORVU.MOREX.r3.6HG0634290 exhibited peak expression in EPI.

GO classification of candidate protein-coding genes (TPM > 100) within SR resistance QTLs revealed distinct patterns in biological processes, cellular localization, and molecular functions (**Supplementary Table 8**, **Supplementary Figure 2**).

The GO analysis revealed that the most enriched biological process categories were related to fatty acid and lipid metabolism, immune and defense responses, and cell wall and structural organization, each represented by 10 or more genes. These results suggested a multifaceted role of metabolic pathways, structural remodeling, and stress signaling in the response to SR resistance. In the molecular function category, the predominant terms were catalytic activity and nucleotide/ATP binding, followed by functions associated with electron transport, glycosylation, and enzyme regulation, reflecting diverse biochemical roles of the candidate genes. For the cellular component category, the majority of gene products were localized to the membrane, macromolecular complexes, and cytoplasmic compartments, with additional enrichment in the cell wall, mitochondrion, and Golgi apparatus.

Overall, the GO annotation suggested that the candidate genes associated with SR resistance QTLs were primarily involved in metabolic and enzymatic functions (especially lipid and carbohydrate metabolism) and were distributed across key cellular structures, including membranes, the cell wall, and the extracellular matrix.

**Figure 8:**
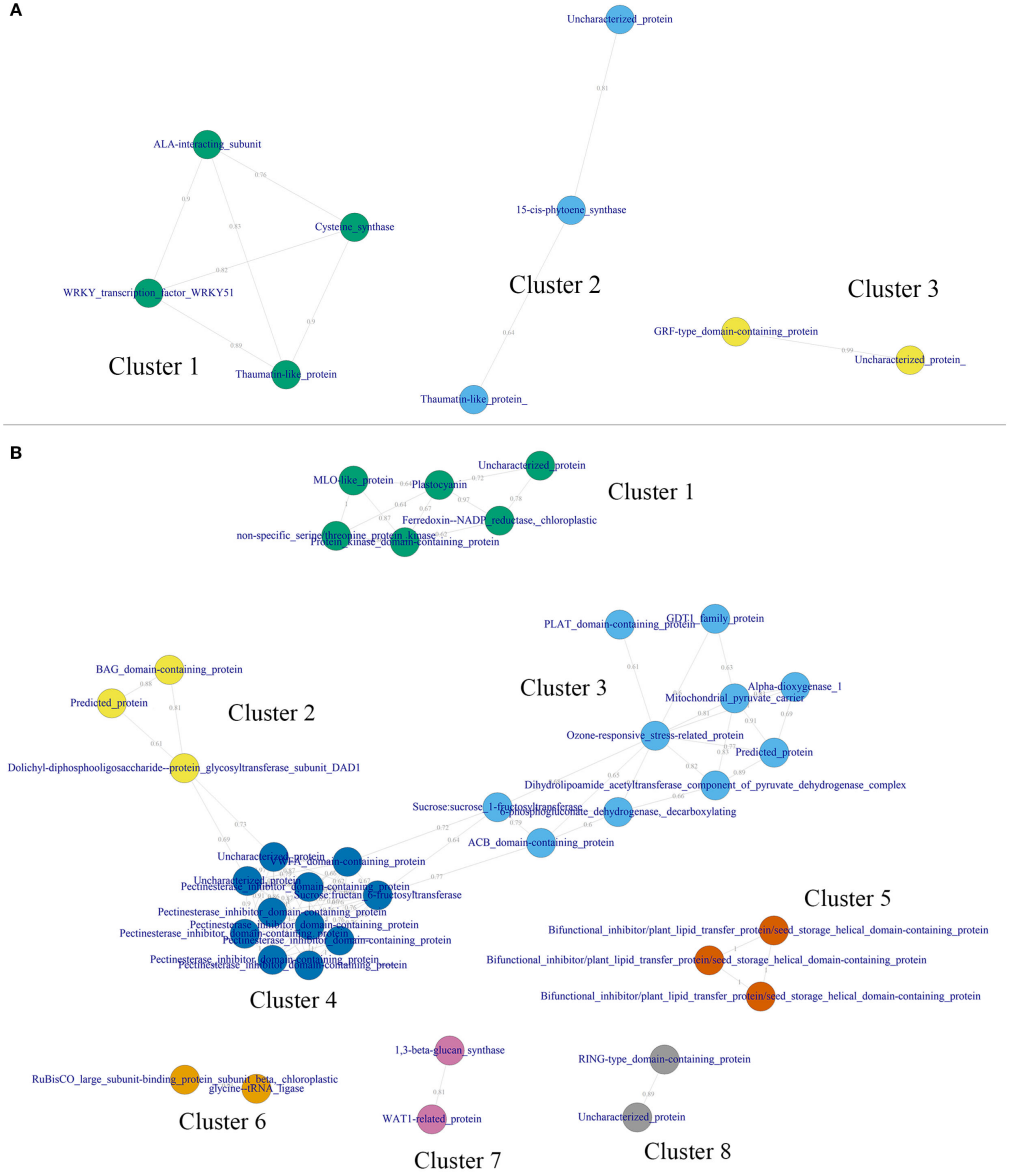
Gene co-expression network for highly-expressed genes (TPM > 100) in QTLs *Q_rpg_5H.1***(A)** and *Q_rpg_7H.1***(B)**. Colored nodes denote genes. Numbers on the edges are Pearson’s *r*-values.

A weighted gene co-expression network (**Figure 8**) was generated based on expression data from 16 tissues/organs to uncover co-expression patterns and identify functionally related gene clusters associated with two stable QTLs – *Q_rpg_5H.1* and *Q_rpg_7H.1*.

For *Q_rpg_5H.1*, three gene co-expression clusters were identified, comprising four (Cluster 1), three (Cluster 2), and two (Cluster 3) genes, respectively (**Figure 8A**). Cluster 1 included genes involved in transcriptional regulation (WRKY51), direct antifungal activity (thaumatin-like protein), membrane transport (ALA-interacting subunit), and supportive primary metabolism (cysteine synthase). Cluster 2 also contained a thaumatin-like protein, along with 15-cis-phytoene synthase and an uncharacterized gene. Cluster 3 comprised a GRF-type domain-containing protein and another uncharacterized gene.

For *Q_rpg_7H.1*, eight clusters were identified, with gene counts ranging from 2 (Clusters 6–8) to 11 (Cluster 4) (**Figure 8B**). Clusters 2, 3, and 4 exhibited strong inter-cluster connectivity, forming a meta-cluster. Cluster 4, the largest, contained genes related to cell wall integrity maintenance, including multiple pectinesterase inhibitor domain-containing proteins and a VWFA-domain-containing protein. Cluster 3, interconnected with Cluster 4, comprised genes associated with a metabolic defense module, encompassing energy production and respiration (mitochondrial pyruvate carrier, pyruvate dehydrogenase, oxidative pentose phosphate pathway), oxidative stress responses (α-dioxygenase 1, ozone-responsive protein, NADPH-generating enzymes), lipid-based signaling (PLAT and ACB domain-containing proteins), calcium signaling and transport (GDT1), and carbon storage and redistribution (fructosyltransferases). The smallest member of the meta-cluster, Cluster 2, included three genes involved in stress-induced signaling and regulation of programmed cell death. Additionally, Cluster 1 consisted of six genes implicated in photosynthetic energy supply, redox homeostasis, and pathogen-triggered signaling, including an MLO-like protein.

Together, the clusters identified within the stable QTLs *Q_rpg_5H.1* and *Q_rpg_7H.1* reveal a coordinated and multilayered defense architecture in barley, integrating transcriptional regulation, antifungal defense, reinforcement of cell wall structure, energy and redox metabolism, lipid-mediated and oxidative stress responses, and regulation of cell death –underscoring their potential roles in enhancing basal and inducible resistance to stem rust.

## 4 Discussion

### 4.1 Phenotypic variation and trait correlations in barley reaction to SR

The evaluation of 273 two-row spring barley accessions across two distinct environments – RIBSP and KRIAPG – revealed substantial phenotypic variation in response to SR, underscoring the genetic diversity of the studied panel. The overall disease severity was lower in RIBSP (mean score 4.4) than in KRIAPG (mean score 5.2), suggesting environmental modulation of *Pgt* development. Differences in inoculum pressure, microclimate, or pathogen race composition between the two sites may have contributed to these disparities (Abdelghany et al., 2024). This is further supported by the variation in phenotypic distributions (**Figures 2A, B**): while RIBSP displayed a near-normal distribution centered around moderate scores (peaking at score 4), the bimodal distribution observed in KRIAPG (peaks at 3 and 7) reflects the interaction between genotype and more contrasting environmental and pathogen-related conditions. In the study, 20 genotypes exhibited an R reaction under field conditions of RIBSP and 14 genotypes – R reaction at KRIAPG; however, such responses are typically conferred by major race-specific genes that, while effective, are often rapidly overcome by pathogen evolution (Michel et al., 2023). In contrast, 119 accessions showing MR and 128 accessions with MS reactions at RIBSP, along with 95 MR and 118 MS accessions at KRIAPG (**Supplementary Table 4**), are of greater breeding relevance. These phenotypes are indicative of partial or slow-rusting resistance mechanisms associated with APR genes. Such resistance reduces the rate of pathogen development without completely preventing infection, thereby providing a more durable and stable defense against stem rust (Michel et al., 2023).

The correlation analysis revealed a significant positive association between PH and SR severity in RIBSP, implying that taller plants were more susceptible (**Figure 2C**). This could be attributed to microclimatic factors within the canopy (Vidal et al., 2017) or differential exposure to inoculum (Araujo et al., 2023). In contrast, a significant negative correlation between TKW and SR severity (**Figure 2C**) suggests that disease burden may adversely affect grain filling and productivity. These findings are consistent with prior studies where rust infections were associated with reduced grain yield and kernel weight due to compromised photosynthate allocation and premature senescence (Junk et al., 2016; He et al., 2019; Zhou et al., 2022a). The low correlations between SR and other agronomic traits suggest that SR resistance is most likely genetically independent, supporting the rationale for performing a separate GWAS for this trait.

### 4.2 Identification of stable and novel QTLs for stem rust resistance

Genotype-based population structure analysis based on kinship and PCA revealed the presence of genetically distinct subgroups within the germplasm collection, likely reflecting differences in geographic origin and breeding history (**Figure 3**). Similar results were obtained previously with similar barley germplasm (Genievskaya et al., 2022, 2023).

The GWAS conducted using five statistical models across two environments led to the identification of 204 MTAs, among which 96 were considered robust and stable due to their detection by multiple models (**Supplementary Table 5**). The highest number of associations was detected using the GLM model, although the MLM and multi-locus models (MLMM, FarmCPU, BLINK) provided more stringent control for confounding factors, thereby enhancing the reliability of identified signals. Haplotype-based consolidation of associated SNPs allowed the definition of 19 model-stable QTLs distributed across all seven barley chromosomes. The QTLs *Q_rpg_5H.3* and *Q_rpg_7H.1* were consistently detected by all five GWAS models in both environments (**Table 1**), suggesting their strong and environmentally stable contribution to SR resistance. Although only a limited number of studies have focused on QTL mapping and GWAS for SR resistance in barley, the current study identified six candidate SR-resistance QTLs and/or *Rpg* genes, confirming their stability not only under Kazakhstan’s environmental conditions but also across other global regions (**Table 2**). Among them, *Q_rpg_2H.4* (617.6–617.7 Mb) was located near previously reported SR-resistance QTLs at 612.5 Mb (Czembor et al., 2022) and 616.4 Mb (Amouzoune et al., 2022). *Q_rpg_5H.3* (453.3–454.4 Mb) overlapped with a QTL reported at 453.6 Mb (Czembor et al., 2022), while *Q_rpg_6H.2* (464.4 Mb) was proximal to an MTA for SR resistance at 471.3 Mb previously identified in Kazakhstan (Turuspekov et al., 2016). Similarly, *Q_rpg_7H.4*, positioned at 602.0 Mb, was close to a known QTL at 606.1 Mb (Czembor et al., 2022).

The major-effect *Q_rpg_7H.1* region encompassed the largest number of linked MTAs (n = 60), which is due to the presence of the strongest gene *Rpg1* in this QTL (**Figure 6**). Position of *Q_rpg_6H.1* matched with the position of recessive *Rpg6* from *H. bulbosum*, however, this QTL demonstrated a minor effect only. The remaining QTLs were mapped to genomic regions not previously associated with known *Rpg* genes and/or QTLs, suggesting the presence of novel loci contributing to barley SR resistance (**Table 2**).

Together, the identification of both known and potentially novel QTLs provides a valuable genomic resource for breeding SR-resistant barley in Kazakhstan and globally. These findings enhance our understanding of the genetic architecture underlying SR resistance in the diverse barley germplasm grown in the southern and southeastern regions of Kazakhstan, supporting targeted improvement efforts under local agroecological conditions.

### 4.3 Transcriptional and functional insights into *Rpg1*-associated QTL *Q_rpg_7H.1*

Plants manage their growth and defend against various environmental challenges through an intricate regulatory network (Li et al., 2020). Using expression data from 16 tissues across developmental stages, a total of 531 candidate genes within 13 of 19 SR resistance QTL regions were initially identified (**Supplementary Table 7**). After filtering for high transcript abundance (TPM > 100), 56 highly expressed genes were identified within five QTL regions (**Figure 7**). *Q_rpg_7H.1* and *Q_rpg_5H.1* were prioritized as QTLs with the largest number of highly expressed genes (n = 42 and 10, respectively) (**Supplementary Table 8**) and highest PVE values (0.3063 and 0.3065, respectively) (**Supplementary Table 6**).

Gene Ontology (GO) enrichment analysis of candidate genes within *Q_rpg_7H.1* (which includes *Rpg1*) revealed significant associations with fatty acid and lipid metabolism, immune and defense responses, and cell wall organization (**Figure 8**). These findings align with recent studies in cereal rust resistance, where coordinated metabolic changes and cell wall modifications – such as lignin-based barriers in rice (Zhang et al., 2025) and the phenylpropanoid pathway in wheat (Liu et al., 2022) – contribute to durable defense mechanisms. The *Q_rpg_7H.1* region formed a meta-cluster (**Figure 8B**) integrating modules related to cell wall integrity (e.g., pectinesterase inhibitors, VWFA-domain proteins), energy metabolism, oxidative stress response, lipid and calcium signaling, and regulation of programmed cell death. This modular defense architecture is consistent with previous findings in barley, where coordinated gene modules have been implicated in resistance dynamics (Yuan et al., 2018). The *Rpg1* gene encodes a receptor-like protein with two tandem serine/threonine protein kinase domains (Brueggeman et al., 2006). Within *Q_rpg_7H.1*, two candidate genes – HORVU.MOREX.r3.7HG0636000 and HORVU.MOREX.r3.7HG0640060, encoding a protein kinase domain-containing protein and a non-specific serine/threonine kinase, respectively – were highly expressed during the SEN developmental stage (**Supplementary Table 8**), coinciding with the peak of stem rust infection. Notably, genes containing pectinesterase inhibitor (PEI) domains from Cluster 4 of the meta-cluster (**Figure 8B**) are implicated in cell wall-based defense and mirror findings at the *Rrs2* locus, where PEI genes co-segregated with resistance to *Rhynchosporium commune* in barley (Marzin et al., 2016), suggesting conserved mechanisms in pathogen defense.

Collectively, the integration of cell wall-associated components (e.g., pectinesterase inhibitors), metabolic pathways (energy metabolism, lipid signaling), oxidative stress response, calcium signaling, and regulators of programmed cell death in the co-expressed clusters of *Q_rpg_7H.1* highlights a robust, multilayered defense strategy. This architecture supports both basal and inducible immunity, consistent with the known mechanism of *Rpg1*-mediated stem rust resistance, which involves early kinase signaling and programmed cell death (Zhang et al., 2008; Shen et al., 2017; Solanki et al., 2019). At the same time, SNPs identified within *Q_rpg_7H.1* in the current GWAS represent valuable markers for marker-assisted selection (MAS) of SR-resistant barley genotypes.

### 4.4 Identification and functional analysis of candidate genes within novel QTL *Q_rpg_5H.1*

The QTL *Q_rpg_5H.1*, identified based on resistance data from KRIAPG, was mapped to a 3.5 – 9.9 Mb interval on chromosome 5H, a region where no previously reported *Rpg* genes or SR resistance QTLs have been described. This QTL includes three linked MTAs detected by all five GWAS models, with *P*-values ranging from 9.73E-04 to 2.86E-08 (**Supplementary Table 6**). Expression profiling revealed 151 protein-coding genes within this interval with available transcriptomic data from 16 barley organs and developmental stages. Of these, 10 genes exhibited high expression levels (TPM > 100) and were designated as candidate genes for *Q_rpg_5H.1*.

Co-expression analysis of these highly expressed genes (**Figure 8A**) identified three distinct clusters, each potentially contributing to SR resistance. The largest, Cluster 1, comprised four genes: a WRKY51 transcription factor, a thaumatin-like protein, an ALA-interacting subunit, and cysteine synthase. WRKY transcription factors are well-established regulators of plant immune responses, orchestrating downstream signaling and secondary metabolism (Sari et al., 2019). Thaumatin-like proteins, classified as PR-5 proteins, possess direct antifungal properties and are typically upregulated upon pathogen attack, including during powdery mildew infection in wheat (Allario et al., 2023). Cysteine synthase is involved in sulfur amino acid metabolism and has been implicated in redox regulation and stress defense, as demonstrated for barley cystatins (Velasco-Arroyo et al., 2018). The ALA-interacting subunit may contribute to membrane transport or signaling processes associated with defense.

Cluster 2 contained a second thaumatin-like protein, 15-cis-phytoene synthase, and one uncharacterized gene. Phytoene synthase is a central enzyme in carotenoid biosynthesis, a pathway linked to reactive oxygen species (ROS) scavenging and signaling during defense responses (Zhou et al., 2022b), though its role in rust resistance in barley remains to be clarified. Cluster 3 consisted of a GRF-type domain-containing protein and another uncharacterized gene. GRF transcription factors, typically associated with plant growth and organ development, are increasingly recognized for their involvement in stress adaptation and environmental response modulation, including in wheat and rice (Cheng et al., 2023). Taken together, these clusters represent a coordinated defense network comprising classical immune regulators (e.g., WRKY, PR-5), metabolic enzymes (e.g., cysteine synthase), and regulatory proteins (e.g., GRF domains). The modular structure of this co-expression architecture – linking transcriptional regulation, antifungal activity, metabolism, and signaling – parallels systems biology models of cereal-pathogen interactions, where network hubs predict resistance phenotypes [for example, *Fusarium* head blight resistance in wheat (Sari et al., 2019)]. These findings suggest that *Q_rpg_5H.1* represents a previously uncharacterized, multi-functional resistance locus with strong potential for MAS and functional validation in breeding for SR resistance in barley.

## 5 Conclusion

Among 273 barley accessions evaluated across two environments in Kazakhstan, a wide range of responses to *Pgt* was observed. A multi-model GWAS approach identified 204 MTAs, among which 96 were considered robust and stable across models, resulting in the delineation of 19 model-stable QTLs distributed across all barley chromosomes. Six of these QTLs overlapped with known *Rpg* genes or previously reported SR-resistance loci, confirming their stability and effectiveness under diverse environmental conditions. The strongest QTL, *Q_rpg_7H.1*, coincided with *Rpg1*, while *Q_rpg_6H.1* co-localized with *Rpg6*. Based on gene expression profiles, major-effect *Q_rpg_7H.1* (*Rpg1*) and the novel major-effect QTL *Q_rpg_5H.1* were prioritized due to the presence of the highest number of highly expressed genes. Functional annotation revealed that *Q_rpg_7H.1* harbors 42 such genes, forming a multilayered co-expression network associated with cell wall organization, lipid metabolism, oxidative stress response, and programmed cell death – processes central to *Rpg1*-mediated resistance. The novel QTL *Q_rpg_5H.1* contained 10 highly expressed genes grouped into three co-expression clusters, including WRKY transcription factors, PR-5 proteins, and regulatory genes involved in defense signaling and metabolism. These findings support a modular, systems-level defense architecture underlying SR resistance in barley. The study enhances understanding of the genetic architecture of SR resistance in germplasm adapted to the southern and southeastern regions of Kazakhstan and identifies valuable targets for MAS in breeding programs. Further fine-mapping and functional validation of *Q_rpg_5H.1* are needed to confirm its causal genes and effectiveness against diverse *Pgt* races, ultimately contributing to durable resistance under variable agroecological conditions.

## Data Availability

All datasets generated and/or analyzed during this study are included in the article and its **Supplementary Material**.

## Supplementary material

Supplementary Figure 1 Single nucleotide polymorphism (SNP) density plot across the seven barley chromosomes.

Supplementary Figure 2 Gene Ontology (GO) annotation of identified genes in barley. The bar plots show the distribution of functional categories across three GO domains: Molecular Function (yellow), Cellular Component (purple), and Biological Process (green). The x-axis represents the frequency (count) of annotated genes in each category.
