# Correction: Dissecting adult plant resistance to stem rust through multi-model GWAS in a diverse barley germplasm panel

**DOI:** 10.3389/fpls.2025.1769859

**Published:** 2026-01-13

**Authors:** Frontiers Production Office

**Affiliations:** Frontiers Media SA, Lausanne, Switzerland

**Keywords:** *Hordeum vulgare* L., disease resistance breeding, quantitative trait loci (QTLs), *Puccinia graminis* f. sp. *tritici*, gene expression analysis

During production, affiliations 1-3 “Laboratory of Molecular Genetics, Institute of Plant Biology and Biotechnology, Almaty, Kazakhstan”, “Laboratory of Phytosanitary Safety, Research Institute of Biological Safety Problems, National holding “QazBioPharm”, Gvardeisky, Zhambyl, Kazakhstan”, and “Laboratory of Cereal Crops, Kazakh Research Institute of Agriculture and Plant Growing, Almalybak, Almaty, Kazakhstan” was erroneously updated to “Laboratory of Phytosanitary Safety, Research Institute of Biological Safety Problems, National holding “QazBioPharm”, Gvardeisky, Kazakhstan”, “Laboratory of Cereal Crops, Kazakh Research Institute of Agriculture and Plant Growing, Almalybak, Kazakhstan”, and “Laboratory of Cereal Crops, Kazakh Research Institute of Agriculture and Plant Growing, Almalybak, Almaty, Kazakhstan”. The corrected affiliations are “Laboratory of Molecular Genetics, Institute of Plant Biology and Biotechnology, Almaty, Kazakhstan”, “Laboratory of Phytosanitary Safety, Research Institute of Biological Safety Problems, National Holding “QazBioPharm”, Gvardeisky, Zhambyl, Kazakhstan”, and “Laboratory of Cereal Crops, Kazakh Research Institute of Agriculture and Plant Growing, Almalybak, Almaty, Kazakhstan”.

The original version of this article has been updated.

