# Supplementary figures and images for: Dissecting adult plant resistance to stem rust through multi-model GWAS in a diverse barley germplasm panel
Source: Front Plant Sci. 2025 Oct 8;16:1681398. doi: 10.3389/fpls.2025.1681398 (PMC12540453; doi:10.3389/fpls.2025.1681398)

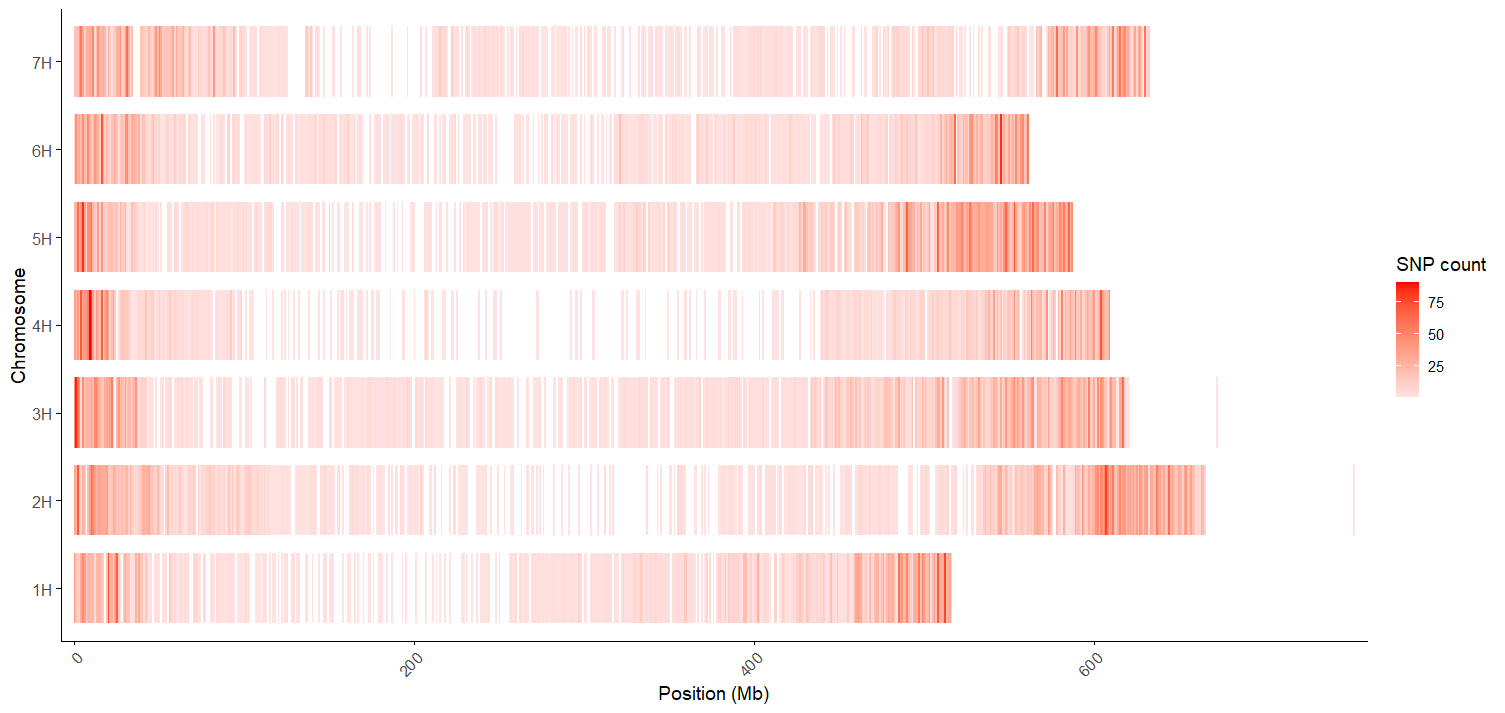

Supplement: Supplementary Figure 1 — Single nucleotide polymorphism (SNP) density plot across the seven barley chromosomes. [file Image1.png]

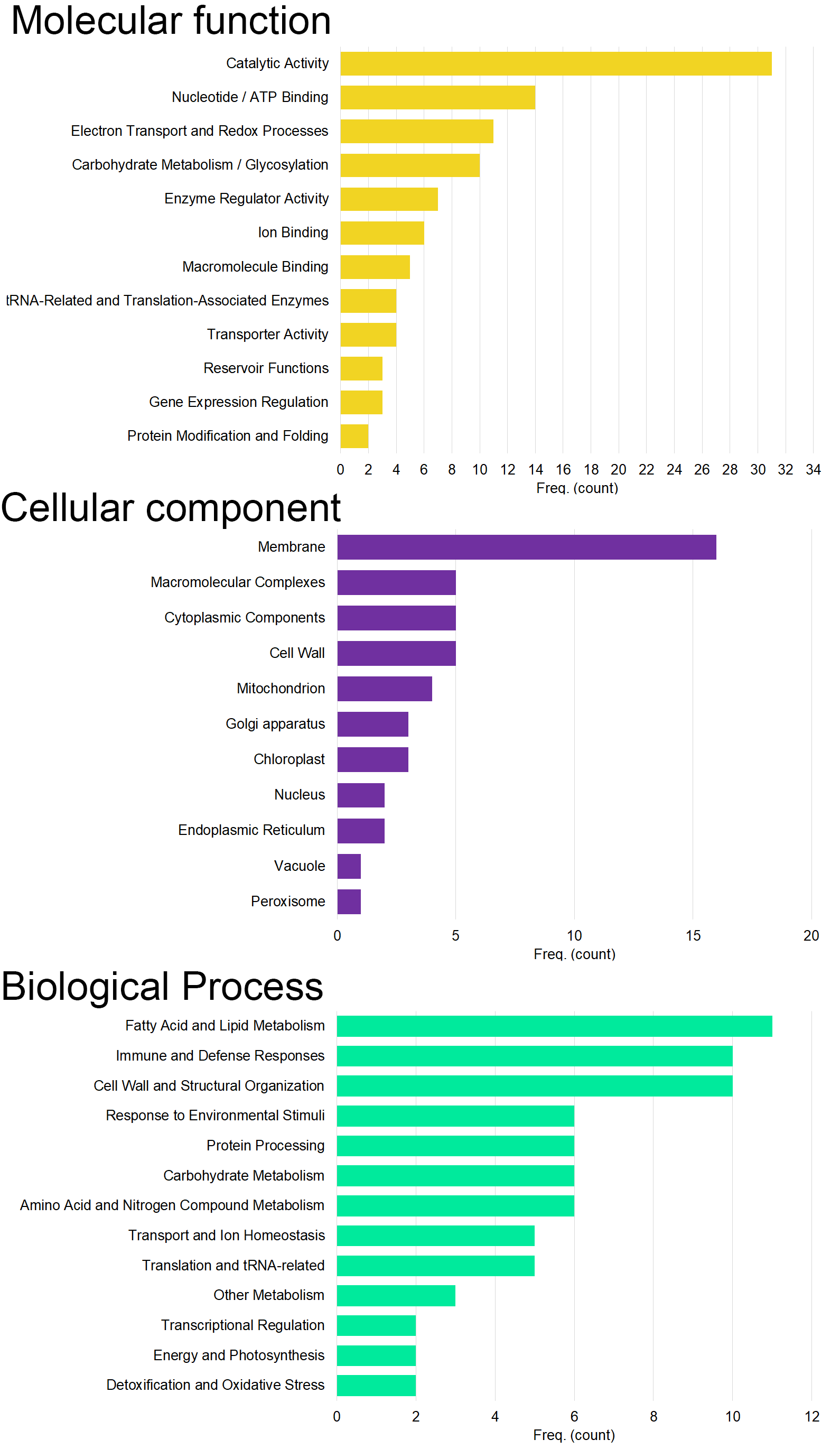

Supplement: Supplementary Figure 2 — Gene Ontology (GO) annotation of identified genes in barley. The bar plots show the distribution of functional categories across three GO domains: Molecular Function (yellow), Cellular Component (purple), and Biological Process (green). The x-axis represents the frequency (count) of annotated genes in each category. [file Image2.png]
