# Supplementary material for: Dissecting adult plant resistance to stem rust through multi-model GWAS in a diverse barley germplasm panel
Source: Front Plant Sci. 2025 Oct 8;16:1681398. doi: 10.3389/fpls.2025.1681398 (PMC12540453; doi:10.3389/fpls.2025.1681398)
Supplement: Supplementary file 4 [file Table2.docx]

**Supplementary Table 2.** Virulence/avirulence formulae of virulent mixture of *Pgt* races used in this study. Virulence lists *Sr* genes against which isolates are virulent (ineffective), and avirulence lists *Sr* genes remaining effective. (*Sr* prefix omitted in the table for brevity.)

| Races | Virulence spectrum (ineffective *Sr* resistance genes) | Avirulence spectrum (effective *Sr* resistance genes) |
| --- | --- | --- |
| QHHSF | *5, 21, 6, 9g, 9b,17, 9a, 9d, 10, 38, Mcn* | *9e, 7b, 11, 8a, 36, 30, Tmp, 24, 31* |
| RFRTF | *5, 21, 7b, 8a, 9g, 36, 9b, 17, 9a, 9d, 10, Tmp, 38, Mcn* | *9e, 11, 6, 30, 24, 31* |
| THMTF | *5, 21, 9e, 7b, 6, 9g, 36,17, 9a, 9d, 10, Tmp, 38, Mcn* | *11, 8a, 9b, 30, 24, 31* |
| RHMRF | *5, 21, 7b, 6, 9g, 36, 17, 9a, 9d, Tmp, 38, Mcn* | *9e, 11, 8a, 9b, 30, 10, 24, 31* |
| TKRPF | *5, 21, 9e, 7b, 6, 8a, 9g, 36, 9b, 17, 9a, 10, Tmp, 38, Mcn* | *11, 30, 9d, 24, 31* |
| MHCTC | *5, 7b, 6, 9g, 17, 9a, 9d, 10, Tmp, Mcn* | *21, 9e, 11, 8a, 36, 9b, 30, 24, 31, 38* |
| RHRTP | *5, 21, 7b, 6, 9g, 36, 9b, 17, 9a, 9d, 10, Tmp, 24, 38, Mcn* | *9e, 11, 8a, 30, 31* |

Literature:

1. Rsaliyev, A., Yskakova, G., Maulenbay, A., Zakarya, K., Rsaliyev, S. (2020). Virulence and race structure of *Puccinia graminis* f. sp. *tritici* in Kazakhstan. Plant Prot. Sci. 56, 275–284. doi: 10.17221/172/2019-PPS
2. Olivera P., Newcomb M., Szabo L.J., Rouse M., Johnson J., Gale S., Luster D.G., Hodson D., Cox J.A., Burgin L., Gilligan C.A., Patpour M., Justesen A.F., Hovmøller M.S., Woldeab G., Hailu E., Hundie B., Tadesse K., Pumphrey M., Singh R.P., Jin Y. (2015): Phenotypic and genotypic characterization of race TKTTF of *Puccinia graminis* f.sp. *tritici* that caused a wheat stem rust epidemic in southern Ethiopia in 2013/14. Phytopathology, 105: 917–928.
3. Bhattacharya S. (2017): Deadly new wheat disease threatens Europe's crops. Nature, 542: 145–146.
4. Lewis C.M., Persoons A., Bebber D.P., Kigathi R.N., Maintz J., Findlay K., Corredor-Moreno P., Harrington S.A., Kangara N., Berlin A., Garcia R., German S.E., Hanzalová A., Hodson D., HovmØller M.S., Huerta-Espino J., Imtiaz M., Mirza J.I., Justesen A.F., Niks R.E., Omarani A., Patpour M., Pretorius Z.A., Roohparvar R., Sela H., Singh R.P., Steffenson B., Visser B., Fenwick P.M., Thomas J., Wulff B.B.H., Saunders D.G.O. (2018): Potential for re-emergence of wheat stem rust in the United Kingdom. Communications Biology, 8: 13. doi: 10.1038/s42003-018-0013-y
